# Spiritual care in outpatient oncology: a qualitative study of focus groups with cancer center chaplains

**DOI:** 10.1007/s00520-025-09369-x

**Published:** 2025-03-26

**Authors:** Kelsey B. White, Petra J. Sprik, Bronwen Jones, George Fitchett

**Affiliations:** 1 Department of Patient Counseling, Virginia Commonwealth University, Richmond, VA USA; 2 Department of Health Services Administration, University of Alabama at Birmingham, Birmingham, AL USA; 3 Cedars-Sinai Cancer Center, Samuel Oschin Comprehensive Cancer Institute, Los Angeles, CA USA; 4 Department of Religion, Rush University Medical Center, Health & Human Values, Chicago, IL USA

**Keywords:** Oncology, Cancer care, Spiritual care, Chaplain, Outpatient

## Abstract

**Purpose:**

To provide a preliminary description of the scope and nature of spiritual care services in outpatient oncology settings.

**Methods:**

Qualitative thematic analysis of data collected from three focus groups with chaplains representing 13 unique cancer centers.

**Results:**

Eight of 13 chaplain respondents (61.5%) reported that they provided spiritual care exclusively and in a full-time capacity to a cancer center; the remaining 5 (38.5%) had additional inpatient responsibilities. Chaplains visited between 4 and 10 patients per day depending on departmental policies and case acuity. Respondents identified patients for care in a wide variety of ways and described it as a time-intensive aspect of their job. Chaplains noted providing traditional spiritual care and developing innovative strategies/techniques. Most spiritual care relationships were long-term and often focused on medical decision-making. Chaplains commonly faced organizational challenges and identified priorities for strengthening spiritual care integration in outpatient cancer care.

**Conclusion:**

The results indicate that the provision of spiritual care in cancer centers differs widely, with chaplains frequently facing challenges with system integration. While chaplains consistently strive to build relationships with clinicians and effectively manage clinic workflows, more collaboration and strategic alignment are needed between chaplains, clinicians, and administrators to develop and advocate for outpatient oncology spiritual care.

## Introduction

Receiving a cancer diagnosis and treatment is a stressful experience for many people. In three large studies of cancer patients the prevalence of distress ranged from 33 to 52% [1–3]. Research describes the importance of religion and spirituality (R/S) in coping with cancer and other serious illness [4–6]; for a majority of cancer patients, R/S is one of the most important resources that help them cope with their illness [7, 8]. A large meta-analysis reported positive associations between R/S and measures of social, emotional, and physical health among patients with cancer [9–11].

Many cancer patients also experience R/S distress or pain. Among cancer patients in general, 19 to 30% report R/S distress [12–14]. Among those receiving palliative care, 44 to 67% report R/S distress [15, 16], with 20% reporting moderate or high levels of distress [17, 18]. Cross-sectional studies among cancer patients find that R/S distress is associated with greater depression, anxiety, pain, fatigue, and poorer quality of life and life satisfaction [17, 19–21]. Two longitudinal studies found higher baseline levels of R/S struggle were associated with more depressive symptoms and poorer quality of life at follow-up [22, 23]. R/S distress has also been described among cancer caregivers [24]. Research finds that cancer patients and their family caregivers welcome spiritual care [25, 26].

The growing attention to outpatient spiritual care [27, 28] comes as hospital spiritual care departments report providing some routine outpatient care, most often in palliative care or oncology [29]. The importance of outpatient spiritual care is increasingly recognized and provided through advancements with spiritual assessment techniques and targeted spiritual care interventions [28, 30]. Improvements in outpatient spiritual assessment demonstrate both feasibility and acceptability [26, 31] and identified the feasibility and acceptability of telephone-based spiritual care [14]. For example, one chaplain intervention delivered to 24 outpatients with advanced cancer and 18 of their caregivers, found improved spiritual well-being at all follow-up timepoints [26]. More than 80% of patients and caregivers reported they would recommend the sessions to others. Targeted spiritual care interventions that have been implemented in outpatient oncology settings have focused on such things as legacy making (e.g., [32]), contemplative spiritual practices, spiritual coaching, and staff care [30]. Another study described spiritual care provided to 27 cancer outpatients, in person or by phone, and its effects [33]. Forty-five percent of the patients had moderate or severe chaplain-assessed spiritual concerns; on average patients had four sessions with a chaplain (range 2–9), and at follow-up, there were decreases in several measures of R/S distress. While these studies attest to the need, desire, and effect of spiritual care in this setting, they do not describe the scope and nature of clinical practice. This limits the ability to establish spiritual care practice guidelines and benchmarks in outpatient oncology settings, as the scope and nature of chaplain-delivered care in these settings is largely unknown.

The aim of the present study was to provide a preliminary description of spiritual care services in the outpatient oncology setting as a step to develop this emerging field of study. Information gained from this study provides critical information about key issues, practices and goals of outpatient spiritual care within oncology settings.

## Methods

### Study design

To explore the staffing, scope, and experience of spiritual care in outpatient cancer centers in North America, the team utilized a qualitative, focus group approach. The Institutional Review Board at Virginia Commonwealth University (IRB No. HM20028065) deemed this study exempt, and thus, the human ethics and consent to participate were not applicable. All participants did consent to participation at the beginning of each focus group.

### Recruitment

Eligible chaplains were those working in an outpatient cancer care setting at least 20% of their clinical time. The study team recruited a convenience sample from their professional networks consisting of chaplains working in cancer centers of diverse sizes, locations, and cancer program designations. A total of 19 chaplains were invited via email and asked to complete a screening survey to ensure they met eligibility criteria and were willing to participate. Two chaplains who completed the screening survey did not qualify and four withdrew after screening; 13 chaplains participated in the focus groups.

### Focus group questions

The research team collaborated with an advisory committee on the development of the focus group questions to ensure content validity and applicability. The advisory committee included medical oncologists, surgical oncologists, radiation oncologists, psycho-oncologists, and chaplains from across the USA. The Appendix includes the interview guide for the semi-structured focus group.

### Data collection

Demographic and professional characteristics were collected in the close-ended screening survey, disseminated by email. Surveys identified the cancer centers where chaplains worked, and a researcher searched cancer center websites to identify designations of cancer centers (National Cancer Institute (NCI), Comprehensive Cancer Centers, and Association of Cancer Care Centers (ACCC)).

Chaplains attended one of three focus groups. Each focus group lasted 90 min and attendees were provided the questions prior to the meeting. Focus groups occurred in November and December 2023 over videoconference software. All four members of the team attended the focus groups and rotated facilitation. Two members would facilitate the conversation while the other two observed and note-took. Chaplain participants were not asked for comment on the transcripts or findings. Focus groups were audio recorded and sent to a third party for transcription.

### Analysis

Chaplain characteristics were obtained from chaplains’ responses to the screening survey, and presented as counts and percentages. For qualitative analysis, team members who observed the focus groups took detailed notes from their observations. Those notes were used to develop a preliminary codebook. Data saturation, defined as the point at which comments and themes are being repeated without the introduction of new substantive information [34], was reached by the third focus groups, and determined using notes taken by the observers.

**Fig. 1:**
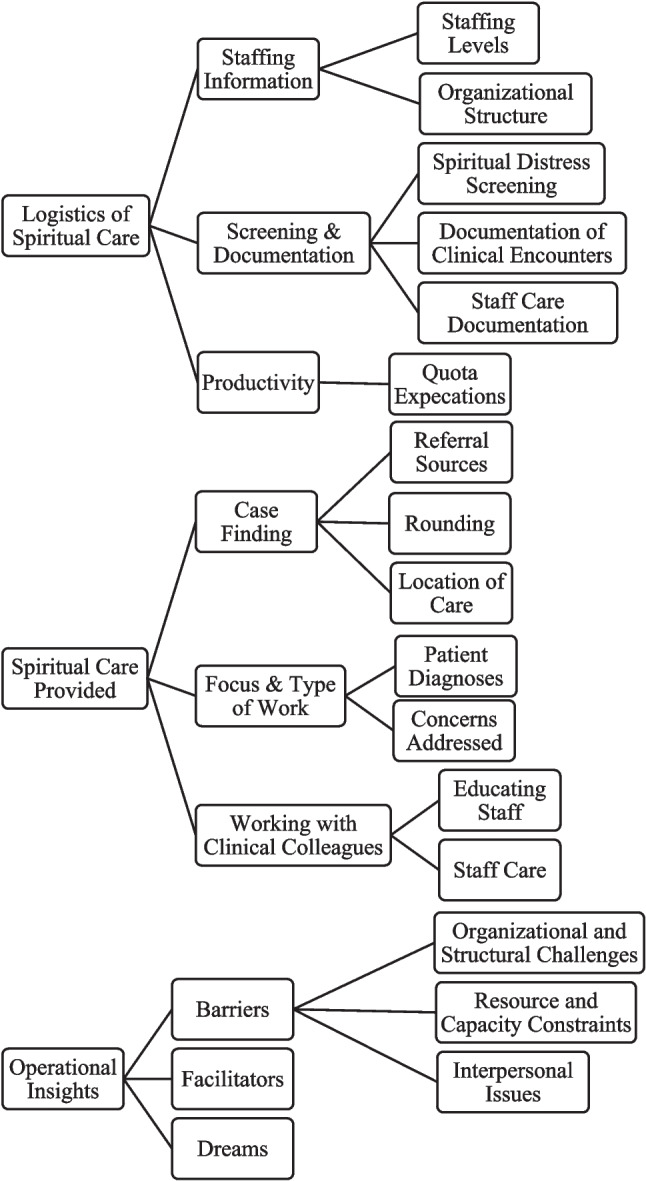
Coding tree

Three coders (KW, PS, and BJ) conducted the thematic analysis [35] and each conducted initial coding of a single focus group transcript. The team then met to ensure the codebook thoroughly captured the patterns within the transcripts (see Coding Tree in Fig. 1). Then, each focus group transcript was coded by a second person for intercoder triangulation, and quality assurance. Discordance was discussed by the team. The team used Qualtrics for screening data collection and Microsoft Excel and AtlasTi for analysis.

## Results

The 13 chaplain participants represented 13 unique cancer centers. These cancer centers operated in the midwest (14.3%, *n* = 2), northeast (14.3%, *n* = 2), south (42.9%, *n* = 6), and west US census regions (21.4%, *n* = 3), and Canada (7.1%). See Table 1 for participant characteristics. Analysis of the focus groups identified three major themes: the logistics of spiritual care, the care provided, and operational insights.

**Table 1:** Characteristics of chaplain participants, *n* = 13

Characteristic		*N*	Percent
Chaplaincy experience (years)	2–5 years	5	38.5
	5–10 years	4	30.8
	> 10 years	4	30.8
Oncology experience (years)	< 2 years	2	15.4
	2–5 years	6	46.5
	5–10 years	4	30.8
	> 10 years	1	7.7
Board certification status	Board Certified Chaplain	9	69.2
	Board Certified Eligible	4	30.8
Gender	Female	10	76.9
	Male	3	23.1
Age	25–44 years	7	15.4
	45–64 years	5	7.7
	65 years +	1	7.7
Ethnicity	White	13	100.0
Religious affiliation	Catholic/Orthodox Christian	3	23.1
	Mainline Protestant	5	38.5
	Spiritual but not religious	2	15.4
	Evangelical Protestant	2	15.4
	Judaism	1	7.7
Cancer center affiliation	National Cancer Institute (NCI) designated + Association of Cancer Care Centers (ACCC) affiliated	8	61.5
	NCI only	1	7.7
	ACCC only	1	7.7
	Unknown	3	23.1

### The logistics of spiritual care

#### Staffing information

Chaplains reported a range of clinical time spent working in outpatient care; 7 reported working more than 80% of their clinical time in outpatient cancer care, while 4 reported 20–40% and 2 reported 40–80%. Eight chaplains (61.5%) reported exclusive responsibilities within the cancer center while 5 (38.5%) were employees of a hospital spiritual care department and were “shared” with the cancer center. Most reported to a manager within the associated health system or nearby hospital (84.6%, *n* = 11) and one reported to a manager within the cancer center itself. Most of their managers held the title “Director” (53.8%, *n* = 7). Table 2 provides direct quotes from chaplains reporting their center’s spiritual care staffing as well as supervisory structure.

**Table 2:** Example quotations regarding the *logistics of spiritual care*

Theme	Exemplary quotations
Staffing information	
Staffing levels	“Because my job is 50% administrative and 50% clinical, I have split with the full-time chaplain so that she focuses primarily on in-person support.” (Focus Group 1)
	“We have two full-time cancer center chaplains, and we also have resource support through our CPE^b^ program. ….. we have two full-time chaplains for seven sites. We’re embedded into … the multidisciplinary support for all of those centers, for all of those infusion centers and radiation and doctor’s offices, oncologist support, which that started as palliative care, and then bloomed into this full multidisciplinary program, which houses the psychiatrists, psychologists, social workers, dieticians, us, on and on.” (Focus Group 3)
	“Because it’s just me, I decide who to follow back and forth. I write myself sticky notes. I do get solid tumor referrals from my inpatient colleagues on surgery.” (Focus Group 2)
	“All the chaplains in outpatient in the form of phone calls, there’s a list that pops up and well, there are multiple lists of patients that we must see, and one of those patients is outpatients, but 90% of those encounters are phone calls. When it comes to a dedicated chaplain that exclusively caters to outpatients, which would be the satellite locations, we don’t have one right now.” (Focus Group 1)
Organizational structure	“I am not owned by the cancer center; I am assigned from spiritual health to the cancer center. They don’t have an outpatient referral that they’re able to give to me through Epic. I get either in-basket, paged, in person, but it’s a very strange experience to be, sort of, I think, the discipline that is not owned by the cancer center.” (Focus Group 2)
	“We as the chaplains are not hired by the cancer centers. We are basically kind of donated by the spiritual care department, which is embedded in the main medical center. But all of our work is fully outpatient at this point.” (Focus Group 3)
Screening and documentation	
Spiritual distress screening	“They [social workers] use an instrument by the National Comprehensive Cancer Network that’s distress management. And on that assessment, if there’s spiritual or religious concerns, fear of death or other existential crises, the social worker gets that intake form and then they pass that on to me to reach out to the patient.” (Focus Group 1)
	“It is a crapshoot as to who actually uses the distress thermometer, the NCCN. So, theoretically, it’s supposed to be being used across the cancer center. They’re supposed to have every patient [complete] it at least once, typically at the first chemo teach. And I have not received any referrals. I keep being told I will receive referrals and again, they’re trying to figure out how to refer to me when they get the results from the distress screening if it shows spiritual distress.” (Focus Group 2)
	“We have a PHQ-9 that’s supposed to be triggered at certain points in the treatment process. I receive very few referral streams.” (Focus Group 2)
Documentation of clinical encounters	“The expectation is that I will create my own encounter, which is a notes only encounter. So, it’s just a free open note that I just do free text in. Whereas inpatient, there is a specific progress note in Epic that we use.” (Focus Group 2)
	“I was part of the team that developed a template for spiritual care so that it worked both inpatient and outpatient and follows the form of referral source, consent, assessment, intervention, outcome, plan, etc.” (Focus Group 2)
Staff care documentation	“We use a program called REDCap to document how many staff encounters we have and what type.” (Focus Group 2)
Productivity	
Quota expectations	“We’re expected to see six to eight on average a day for full-time, if you’re full-time clinical. I don’t think that that is always doable on the outpatient side because there’s so much of trying to catch patients when they’re coming in and the burden of chart review on the outpatient, it’s just so much higher. So I think that number should be lower. If there were more of us and we were fully integrated onto each disease team, that would be different. But we don’t have rounds to go to, so we have to do a lot more reviewing of charts and running round.” (Focus Group 1)
	“Productivity. Some days I can visit 20 people, but they’re 10-min check-ins, and then sometimes there are 4 people and I’m spending an hour with them because there’s a big change and they really need somebody to sit with them during that time. So I think every day really does differ.” (Focus Group 3)

^a^*FTE*, full-time equivalent; ^b^*CPE*, clinical pastoral education. This is the training required post-graduate degree for those seeking to become professional chaplains

#### Screening and documentation

Several chaplains reported that their centers conducted spiritual distress screening with the National Comprehensive Cancer Network (NCCN) distress thermometer while others used the Patient Health Questionnaire (PHQ)−9. The screening process was primarily administered by other clinicians who assessed whether to refer to spiritual care. At two sites, nurses conducted the screening. Referrals were received either through the electronic medical record or in person. Some reported poor communication and follow-up from the screening process and expressed concerns that potential referrals were never placed.

Chaplains reported on documentation approaches for both clinical encounters and staff care. For clinical encounters, about half the chaplains documented patient encounters within an established template while the other half used free text. Several chaplains reported having their documentation audited by supervisors to garner a total number of visits, rather than for content. Few, mostly more recently trained chaplains, reported that their supervisor assessed content of their charting. While uncommon, some cancer center chaplains tried to document staff care including one that used REDCap (Research Electronic Data Capture—a data collection software) to facilitate record keeping.

#### Productivity

Chaplains noted variation in expectations for productivity (the number of encounters per day); some reported no expectations while others ranged from 4 to 10 patient encounters. Auditing processes that focused on numbers of visits tended to encourage chaplains to bolster numbers by cold-calling patients (visiting without a referral) in the infusion area to meet the quota, with visit lengths at about 10 min. The chaplains indicated that meeting quotas had a direct impact on the quality of care; focusing on numbers detracted from being able to forge deep connections. Several chaplains complained that the expectations for the number of patients seen per day were the same for inpatient and outpatient spiritual care. All agreed that locating patients in the outpatient setting was often time consuming and unpredictable; they believe that expectations for outpatient work productivity should be lower than for inpatient work.

### Spiritual care provided

The chaplains discussed several factors related to the nature of their spiritual care (see Table 3). Specifically, they reported on “case finding” (identifying patients for spiritual care), the focus of care, location, and type of care provided. Their integration relied heavily on educating clinical colleagues.

**Table 3:** Example quotations regarding the *spiritual care provided* by chaplains

Theme	Exemplary quotations
Case finding	
Referral sources	“A nurse will page me from infusion and ask me to come and there’s rare occasions where I’ll attend a palliative care session with a patient or some other meeting. And I do get referrals as well from the outpatient palliative care team.” (Focus Group 2)
	“Most of my time, in all honesty, does not come from referrals. It comes through intentional rounding in the infusion center. And after that, I would say I try to be present in the waiting areas. And then following that, there may be some direct referrals that come from nursing staff, CMAs. Sometimes the providers through our electronic health system are paging to go see somebody in the clinic for radiation oncology. And then following that, I would say our distress screening, which I know we’re getting into later. It’s not a spiritual screening.” (Focus Group 3)
Rounding	“In one of the smaller clinics, I do some of that cold calling and rounding, sort of, check in with nurses to see if any of their patients might benefit from a check-in. I crave more rounding and relational referrals.” (Focus Group 2)
Location of care	“We do a ton of virtual care. I do a ton of virtual care. Most of that is phone call, I think patients really like to be on the phone for some reason. But we also do video visits. I consider video calls or phone calls to be just all telehealth, tele-chaplaincy for me, and basically, I give those patients an option when we’re establishing support. Do you want to do phone calls? Do you want to do video visits? What would you prefer? Most of them choose phone calls, some of them choose video visits, and go from there. Those patients are regular support that I’m providing.” (Focus Group 3)
Focus and type of care	
Patient diagnoses	“The team that tends to refer to me outpatient from the solid tumor side is GI and that’s because there’s two champions nurse practitioners from that team, including the head of survivorship. The other solid tumor patients I see will be sort of random.” (Focus Group 2)
Concerns addressed with spiritual care	“So often a huge part of what I’m doing is about assessing someone’s ultimate priorities, values, beliefs and goals, and then helping with how that translates to their care. And something that I’ve never worked in oncology outside of the current setting that I’m in, but my understanding is that it is not remotely limited to the clinical culture at my cancer center, but really oncology worldwide, is that patient centered care around goals is not the norm. That they sign up for the ride when they start establishing care, and if the oncologist says more radiation, or more chemo, or more surgery, or none of those things, then that’s what happens.” (Focus Group 3)
	“I try to find out what is helping them currently cope. I want to assess their coping skills, or what has helped them in times past when they’ve gone through difficult seasons in their life. My goal is that they share what their strengths are, what their resources are, whether it’s family or if they have a meditation practice or journaling. I want to invite them to think about what’s helped them, that those same tools, those same things that have helped them in previous seasons of their life can also help them through this.” (Focus Group 1)
Working with our clinical colleagues	
Educating staff	“We have a newsletter that goes out from patient family support program and there is a column for spiritual care within that. And then I think otherwise staff knows about us by word of mouth or simply by seeing us and the fact that we sit with other staff members. […] We do education through patient family support program through the staff meetings. We have scheduled education days for each discipline within the patient family support program and chaplaincy is one of those. We provide a case study and then we also provide an education and in the past we’ve done what does it mean and how do you do a spiritual assessment? Why would you refer to a chaplain?” (Focus Group 3)
Staff care	“We provide both formal and informal staff care. So some of our formal staff care is through what we call Tea for the Soul. […] And we go through a list of patients who’ve died in the last three to four months and allow staff to reflect and engage about those patients. It provides an opportunity for memorial and a small bit of time for some grief sharing and things like that […] Our informal staff care is simply that we build relationships with the staff and we often have staff pull us aside or request to chat or just need to process something. Those things happen daily, and it might just simply be that when we’re walking through the infusion center or because we have other infusion sites, I’ll get pinged on Microsoft Teams or by email or a phone call and we share offices [with other clinical team members]. The fact that we’re simply there with those teams makes for casual check-ins with them.” (Focus Group 3)

#### Case finding

Chaplains identified patients to visit through referrals and rounding in clinic areas. The chaplains primarily identified patients/families through verbal referrals from the multidisciplinary team or automated referral mechanisms. Outpatient nurses, social workers, and inpatient chaplains verbally made referrals or communicated those referrals via email. Chaplains also received referrals though automated triggers within the electronic medical record. Many discussed the challenge of how to increase referrals, while others noted that they were too overburdened by current work to identify new referrals.

The location of chaplains’ visits varied, but primarily occurred in infusion centers/areas, clinic rooms/specific clinics, and in private offices/consult rooms. Chaplains also frequently reported conducting telephone or virtual visits. They also provided spiritual care in radiation therapy, pre-surgical areas, and public areas.

#### Focus and types of care

The focus of chaplains’ care did not directly depend on disease type. They cared for patients with solid tumors and blood cancers, with some chaplains specializing or providing more care to patients with specific cancer diagnoses (e.g., breast or prostate cancer). Many newly diagnosed patients were referred to chaplains and met with the chaplain primarily during infusion treatment or over the phone. Chaplains also described the focus of their care as it related to emotional distress. For instance, one chaplain said, “As[patients] are experiencing anxiety following mammograms or around biopsies, that’s often my first meeting.”

The concerns addressed ranged from emotional support, teaching coping techniques, prayer, spiritual counseling, connecting to resources, and companionship. More structured programming included spirituality groups and bereavement groups. Many spiritual care relationships were long-term in nature. Chaplains often provided guidance regarding medical decision-making, such as advanced care planning and other opportunities for patients to talk through the alignment (or misalignment) between their care plan and their beliefs, in one-on-one care encounters and team meetings.

#### Working with clinical colleagues

Chaplains often educated clinical colleagues on/about spiritual care. Specifically, they taught about the chaplain’s role, the scope of spiritual care, role clarity between the chaplain and other psychosocial workers, and their capacity to care for staff.

Chaplains also included staff care within their purview. Some chaplains offered structured programming targeting cancer center employees’ well-being. These programs addressed coping, grief, and difficult clinical experiences. An example is Tea for the Soul (see [36]). Some offered memorial services or other grief-processing opportunities. Unstructured and informal staff care focused on building relationships through informal clinical rounding, regular email communication, personal communications (e.g., sending birthday cards), and individual crisis counseling for socio-political trauma (e.g., Israeli-Palestinian conflict). Successful staff care depended on the strength of established relationships and being “known.”

### Operational insights

Chaplains identified several barriers in their efforts to provide spiritual care, facilitators, and dreams for spiritual care. See Table 4 for exemplary quotations.

**Table 4:** Example quotes regarding operational insights

Theme	Exemplary quotations
Barriers	
Organizational and structural challenges	“My supervisor is a psychologist and our psychology program has a three-month wait list for new appointments. And the goal generally is more patients with appointments. I think that outpatient model, I’ve experienced it to be a little tricky for spiritual care. Most of the patients that I visit with, they don’t want to schedule a phone call with me, but they like it when I call every couple of weeks and catch them at a good time and chat for 30 minutes.” (Focus Group 2)
	“I don’t think that that is always doable on the outpatient side because there’s so much of trying to catch patients when they’re coming in” (Focus Group 1)
	“So the main barrier to getting to the satellite locations and to providing more outpatient is the fact that we have to drive long distances to get to those places. I mean, the main difficulty of getting a chaplain to do outpatient full time is they’re going to have to do a tremendous amount of driving.” (Focus group 1)
Resources and capacity constraints	“That being said, we don’t have any metric for outpatient ratio. When I started, my colleagues and I, we were charged with coming up with the [model] for the outpatient setting and we couldn’t do it. So how do you prioritize?” (Focus Group 2)
	“We have a lot of administrative duties…which include education, which includes staff care, which include, we are responsible for our own calendar management, all of those things, finding our patients, finding the referrals, all of that, which take time out of our schedule.” (Focus Group 3)
	“Funding. Our sister hospital’s issue was going through massive cuts in their system, whereas our system was a much healthier budget and much more value being a Catholic healthcare institution that we retained our spiritual care support and were able to extend it to those patients beyond sometimes the discharge point in our system. And we name that as compassion as being part of our values in our system. But I think it really came down to social work can do this work and we don’t have the budget and what’s the difference of one person here? So, that position was eliminated.” (Focus Group 2)
	“Here, I’ve watched everybody double. We had one social worker, then we had two. We had one PCRM and then we got four. You know what didn’t change? Chaplaincy.” (Focus Group 2)
	“We don’t have something like that [a welcome/informational packet] because we have so many different, each disease team operates as its own little unit, and so they all do things differently.” (Focus Group 1)
	“I will be sitting in my cube in an area where other people are talking, which is not ideal, but I don’t have my own office space when my phone calls” (Focus Group 1)
Interpersonal issues	“Some people think of chaplain equals Christian or chaplain equals religion. And I feel like that’s a disservice; that we can support people of faith and people of no faith.” (Focus Group 1)
	“That was partly what led to the dismissal was a lot of turfiness between social work and spiritual care and a sense that social workers could do what we do. And so, that was what largely eliminated the position of spiritual care.” (Focus Group 2)
	“The parallels of we all feel like we’re working in our own little bubbles, our silos, misunderstood, constantly interpreting, and yet we’re all coming up with a lot of the same... To the same places. We’re all making those same discoveries. And it’s part of me that loves when we share those because I think there’s strength in that kind of networking of, okay, so we don’t have to each of us recreate that wheel.” (Focus Group 2)
Facilitators	
Advocates and supporters of outpatient spiritual care	“One of the incredible benefits that we have upper management who really understand the benefits of spiritual care. So the director of our patient family support program is very supportive of spiritual care. The vice president who oversees spiritual care within the medical center is very supportive of spiritual care. So even with the fact that we come up against budgeting issues and all of that, we do have people in leadership who are supportive of us, understand the work that we do and are champions of that work.” (Focus Group 2)
Integration	“I’m lucky to be part of a broader supportive oncology team. And so, we’ll send out all-staff emails that say, “Reminder about our services,” or there’s posters all over the place or we have a robust, there’s people that will refer to me. If you know about one of the disciplines, you sort of therefore get to be exposed to the others.” (Focus Group 2)
Multiple modalities	“I think for outpatient, it’s critical to have multiple modalities. You can’t just rely on in-person or just rely on telephone. You have to be able to be flexible about that.” (Focus Group 1)
Small practice setting	“I feel very blessed that I have a small group. It’s like 25 to 30 team members... They know me. I know them. And so they feel comfortable just coming to me with whatever they need.” (Focus Group 3)
Standardized interventions	“I think the particularity of a chaplain intervention helps people know what kind of bucket to put the referral in….If you have depression or anxiety, you go to psych. And if you have existential concerns or legacy whatever, just having a particular kind of care helps get more of the right people toward me.” (Focus Group 2)
Dreams	
Automated screening and referral systems	“I really wish we had input to getting more integration through the electronic health record about actually making referrals, making them consistent so that we can have a better opportunity to also sort of prove what we’re doing. And then I like the idea, but there also needs to be time and resources to have more educational opportunities from a interdisciplinary setting to continually coach on different ways that we can be a support to the team and at least at our setting. But those are three things that I think I would personally like to work on, and I think would help further integrate within the system if those things were a priority.” (Focus Group 3)
Better patient education about chaplain’s role	“I would love some sort of standardized education. You’re a new patient, here are all the different services that we provide with spiritual care included.” (Focus Group 1)
Decisional involvement	“We had no voice at the table. It feels like Hamilton. I want to be in the room where it happens so that there can be more advocacy where the decisions are being made. And I’m not saying that that needs to be me as a run-of-the-mill chaplain, but how do we get a voice there that does advocate for spiritual care, spiritual health?” (Focus Group 2)
Improved outpatient chaplaincy training	“And in terms of the CPE piece, and it’s interesting because the past two years I’ve been integrating CPE to outpatient context and that’s a whole other mess of learning and trial and error and pieces that are working, and pieces that aren’t. But it’s really that constant effort of translating inpatient, outpatient, the CPE is designed for inpatient and how do I translate that?” (Focus Group 2)
Earlier care integration	“I would say one [chaplain] in each clinic that can do each of the rounds and be totally invested, go to the [clinic events], go to the relationship building, and then you would be able to follow a patient when they’re diagnosed all the way through to the end. And I think being able to have that would be the most ideal.” (Focus Group 3)
More advanced interventions	“The one other thing I hear is longing for things like dignity therapy and meaning centered group psychotherapy. Folks want to wrestle with legacy. I have the most success when I say, “Hey, this is the thing that I do,” and getting referrals that way, that’s what’s worked. And so I think there’s need for more of that, or there’s longing for more of that particular intervention based care.” (Focus Group 2)
Sustainable work	“And so I want to do this over time. I want to make a difference. I want to be here for patients and families and staff, and I want to make sure that I can sustain myself in doing this work and hopefully be part of sustaining my peers who do this as well.” (Focus Group 2)

#### Barriers

##### Organizational and structural challenges

Chaplains struggled to navigate the expectation that outpatient chaplaincy should function like inpatient chaplaincy. For instance, one chaplain could not visit the established volume quota—an expectation that was established based on inpatient chaplaincy data. Outpatient workflow differs compared to inpatient workflow; administrative and programmatic responsibilities also differ. Outpatient care occurs much like in-depth pastoral counseling at times.

Chaplains also struggled to navigate the expectation that outpatient chaplaincy should function like other outpatient services. While many centers wanted chaplains to schedule appointments with patients like other psychosocial services, patients preferred conversation at pre-existing appointments (like in infusion) rather than to schedule additional appointments. Short appointment times further complicated the provision of spiritual care, as it was difficult for chaplains to find and provide care for patients in short, interrupted appointment time frames.

Covering multiple clinics interfered with chaplains’ abilities to develop staff relationships and be available for patients. Additionally, some reported having limited access to relevant clinical information which limited their ability to identify patients who might need spiritual care. Chaplains described reporting to non-chaplain supervisors whose limited understanding of their role reinforced the chaplains’ perception that spiritual care was a low organizational priority. Inconsistent or unclear organizational processes for spiritual distress screening also made providing spiritual care very challenging. If an organization used a referral system, it often did not function well or the chaplain never received the referrals.

##### Resources and capacity constraints

Chaplains struggled to access sufficient funding for programming, physical space, and limited workload capacity. Many departments had limited or no designated funding for staff-care or patient-care programming. Others did not have a designated physical space which impacted privacy for patient visits (even telechaplaincy visits). Chaplains had no data-driven benchmarks to advocate for positions or structure their own position. This was amplified by training deficits; chaplains’ education had not prepared them for the unique challenges or aspects of outpatient work.

Most of the chaplains were the only chaplains in their center and reported they could not meet the demand for spiritual care. Outpatient chaplains reported juggling more administrative duties such as scheduling appointments, conducting programming, and chart-review than required for inpatient spiritual care. As clinic volume has grown, some chaplains could not keep up, while a few added positions to meet the increased demand. They also struggled to keep up with clinical staff turnover, which served as an impediment to staff awareness of spiritual care.

##### Interpersonal issues

In each focus group, chaplains described feeling isolated, without chaplaincy colleagues with whom they could debrief, collaborate, and network. Chaplains, albeit with different levels of professional experience, felt they needed to prove their value and function, especially with other providers and within their institution.

#### Facilitators

Chaplains identified five key facilitators for their outpatient chaplain work. First, chaplains who had an advocate for outpatient spiritual care found integration and spiritual care provision easier. Advocates, often physicians, lead nurses, or administrators, would support and find funding, educate others about the importance of spiritual care, and provided frequent or quality referrals to the chaplain. Second, chaplains thrived if they had strong supportive oncology departments, palliative care departments, or mental health triage systems. Third, use of multiple modalities to provide spiritual care (telechaplaincy and in-person visits) helped. Fourth, chaplains benefited when assigned to smaller practice settings with manageable workloads and fewer staff with whom to build trusting relationships. Finally, training in providing standardized interventions, such as legacy interventions or dignity therapy, equipped chaplains to provide spiritual care and helped their colleagues better understand chaplains’ care.

#### Dreams

Beyond hopes for improvements in identified barriers, chaplains also identified their hopes for growth in outpatient spiritual care (see Table 4). Many simply wanted enough chaplaincy staff to meet the clinical demand. Some mentioned wanting to strengthen the provision of spiritual care across the entire care continuum or be integrated earlier into the patient’s course of care. One chaplain shared,

I think if the sky was the limit, I would embed a chaplain at every one of our sites and I would give each site a chaplain and an [chaplain] intern […] and put a meditation room at every site. There would be programmed staff care at each site […] At the same time, we would be our own specific team, spiritual care team, and doing virtual huddles as a spiritual care team, seeing what’s working, what’s not working.

Unfortunately, this is not the type of care provided within most cancer centers.

## Discussion

Researchers have identified the importance of attending to spiritual health during serious illness [4] and national organizations’ have emphasized the need to identify spiritual distress early in the care continuum [37]. However, very few studies have documented the extent and nature of spiritual care in outpatient oncology. The present study described the scope of spiritual care staffing and services through focus groups with chaplains working at least 20% of their clinical time in an outpatient oncology setting. Through those focus groups, we identified inconsistent staffing patterns and scope of services. Spiritual distress screenings and case-finding processes manifested in a variety of ways often leading to additional administrative burden for chaplains. The ongoing need for education, and provision of emotional and spiritual support to clinical colleagues further strained chaplains’ efforts to ensure their robust integration in their center.

Two prior studies of psychosocial services in cancer centers have inquired about spiritual care services, but unfortunately, they provide limited information. In a study of 20 NCCN centers [38], they assessed the number of chaplains using categorical variables, including the category of 0–2 chaplains; from this, it is impossible to tell whether there were any chaplains at the 7 centers who selected this response. Additionally, eight of the centers failed to answer this question. The second study surveyed member of the American Psychosocial Oncology Society (APOS) members about services provided at their centers [39]. Three-fourths of the centers reported spiritual care services were available without any additional information about the scope of those services. The findings from the present study suggest there is wide variation in the level of chaplain integration and the services they provide in outpatient cancer centers, if it is offered at all. As R/S care is associated with improved physical, mental, and spiritual coping for cancer patients, organizational leaders should revisit how they prioritize spiritual care integration. Consistent with much [6, 12], but not all [7] current research, this paper focused on spiritual care services for cancer clinic patients in general. Inquiring about spiritual care services for patients from important racial/ethnic or spiritual identities was outside the scope of our work; it remains an important priority for the future research. Further, future research should explore the role healthcare administrators play in navigating the financial opportunities and challenges of more robust spiritual care integration.

Our findings highlighted a gap between more broadly accepted clinical goals and actual organizational implementation. Specifically, spiritual distress is required as part of routine distress screening according to certain certifying bodies [40]. However, chaplains identified that how cancer centers screen for spiritual distress varies widely and how distressed individuals are referred to them is unreliable in many instances. Further, many of the centers use the distress thermometer, a visual analog scale where a patient may indicate their distress from 0 to 10. In prior research, it was not an effective method to screen for spiritual distress [13]. This study adds further concerns about the feasibility of the distress thermometer in clinical practice; specifically, whether this measure gets routed to the chaplain, even if both sections are filled out to indicate spiritual distress. It is possible that another tool would create clearer clinical pathways for referral.

The way these chaplain respondents described their care for and education of their clinical colleagues aligns with existing literature. Whether it is written into formal job descriptions or done informally, chaplains describe spending upwards of 30% of their time caring for colleagues [41]. Additionally, chaplains face a need to constantly educate their peers about spiritual health and their role [42]. Efforts to enhance clinicians’ understanding of spiritual health and identification of distress signals has spawned spiritual generalist trainings [43] and models of interprofessional education in palliative care [44]. Chaplains noted that partnerships with clinician champions and spiritual care advocates also helped further their integration. Future research could consider identifying the aspects of those partnerships that best enable organizational integration as well as whether specific clinical disciplines are best trained to conduct the initial spiritual screening.

### Limitations

While this project uniquely captures the scope of spiritual care staffing and services in cancer centers, it is not without limitations. First, the study employed a convenience and purposeful sample from a network of known outpatient oncology chaplains. We do not know the extent to which this represents all chaplains working in outpatient oncology and thus we cannot generalize from this study. We believe it provides a solid foundation for further investigation. Second, gathering information in a focus group setting can introduce social desirability bias. The facilitators attempted to limit this bias by using rapport to challenge indications of groupthink and asking questions of specific individuals who appeared less engaged [45]. Third, we did not ask about the perceived benefit of spiritual care. For example, we did not ask about the benefits of initiating spiritual care encounters according to the setting nor ask if the mere availability of a chaplain in a waiting room altered the atmosphere. Additionally, our study did not ask about the perceived benefit of staff support. Future research should explore the benefits of the increased awareness of chaplain availability on patients, families, and staff in outpatient cancer care. Finally, this study does not include the important perspectives from chaplains’ clinical colleagues or chaplaincy managers.

## Conclusions

Clinicians and researchers increasingly recognize the importance of screening for spiritual distress and spiritual care within cancer care. The evidence suggests such attention impacts both the utilization of health services and individual-level outcomes, such as coping, quality of life, and health outcomes. According to the chaplains in our study, spiritual care in cancer centers varies considerably and they often struggle with system integration. Chaplains consistently aim to develop partnerships with clinicians and navigate clinic workflows. Given the current model for spiritual care in healthcare, chaplains and spiritual care champions should explore innovative techniques and strategic alliances for improved integration.

## Data Availability

The data that from the present study are available from the corresponding author, but restrictions may apply to the availability of these data to ensure maintenance of participant anonymity.

## Appendix. Focus group questions

**Spiritual Care in Cancer Care**

Focus Group Interview Guide

[Review preamble consent.]

**Does anyone have any questions?**

- Yes – Answer questions or refer to PI.
- No – Continue with the focus group.

Thank you! Before I ask any questions, let me set some ground rules. First, how we each do chaplaincy in each of our settings will differ and we do not expect you to all agree on how to provide spiritual care in outpatient cancer settings. If you have an experience or thought that differs from the rest of the group, we still would like to hear from you. Second, please don’t talk over one another – I know it can be difficult on video conferencing, but let’s try our best. Finally, if we haven’t heard from you or we have heard from you too much, we may invite you to speak or ask you to give others a moment to share. Finally, we ask that you respect each person’s privacy and the content of what they share today. Can we all agree that what is shared today remains here?

If yes, proceed. If no, then ask the individual to leave if they can’t agree upon focus group guidelines.

1. Could each of you briefly describe the setting where you work?
  - How long have your institutions had a chaplain?
  - Do any of your settings employ more than one chaplain?
2. Can you describe the chaplaincy / spiritual care services provide to patients?
  a What kinds of patients are the primary recipients of spiritual care?
    i. Where do you visit the patients (Infusion center, clinic rooms / appointment rooms, imaging, other consultation rooms, waiting areas, etc.)?
  b How do you get referrals or know which patients to see?
  c Do chaplains who work in outpatient visit patients in the inpatient setting for continuous care?
3. What should we be doing (that is not happening) for outpatient spiritual care in the cancer care context?
  a What have been the facilitators in getting closer to that ideal?
  b What have been barriers in spiritual care provision?
4. Does your center use a formal spiritual screening process?
  a What is it?
    i. [Probe for what type of instrument or tool or specific questions they use for screening.]
  b Does it trigger a referral to spiritual care?
    1. If so, how is that referral communicated?
5. How do you most frequently engage with staff?
  a Could you describe the type of spiritual care you provide most frequently to staff?
  b How do the staff at your center learn about spiritual care services and their availability?
6. Most chaplains within healthcare setting are required to document spiritual care. What does the documentation process for spiritual care services look like in your setting?
  a If they primarily talk about patients, ask if they document any of the spiritual care provided to staff.
  b Is there any regular audit of your services or documentation?
    i. Does anyone (administration or supervisor) look for specific outcomes of your services?
7. What else would you consider core to the scope of spiritual care services in outpatient cancer care that we may not have discussed?

## References

[1] Carlson LE, Zelinski EL, Toivonen KI, Sundstrom L, Jobin CT, Damaskos P, Zebrack B (2019) Prevalence of psychosocial distress in cancer patients across 55 North American cancer centers. J Psychosoc Oncol 37(1):5–21. 10.1080/07347332.2018.1521490 30592249 10.1080/07347332.2018.1521490

[2] Mehnert A, Hartung TJ, Friedrich M, Vehling S, Brähler E, Härter M, Keller M, Schulz H, Wegscheider K, Weis J, Koch U, Faller H (2018) One in two cancer patients is significantly distressed: prevalence and indicators of distress. Psychooncology 27(1):75–82. 10.1002/pon.4464 28568377 10.1002/pon.4464

[3] Wang GL, Cheng CT, Feng AC, Hsu SH, Hou YC, Chiu CY (2017) Prevalence, risk factors, and the desire for help of distressed newly diagnosed cancer patients: a large-sample study. Palliat Support Care 15(3):295–304. 10.1017/S1478951516000717 27697082 10.1017/S1478951516000717

[4] Balboni TA, VanderWeele TJ, Doan-Soares SD, Long KNG, Ferrell BR, Fitchett G, Koenig HG, Bain PA, Puchalski C, Steinhauser KE, Sulmasy DP, Koh HK (2022) Spirituality in serious illness and health. JAMA 328(2):184–197. 10.1001/jama.2022.11086Canada2019 35819420 10.1001/jama.2022.11086

[5] Canada AL, Murphy PE, Stein KD, Alcaraz KI, Fitchett G (2019) Trajectories of spiritual well-being in long-term survivors of cancer: a report from the American Cancer Society’s Studies of Cancer Survivors–I. Cancer 125(10):1726–1736. 10.1002/cncr.31967 30633818 10.1002/cncr.31967

[6] Palmer Kelly E, Paredes AZ, Tsilimigras DI, Hyer JM, Pawlik TM (2022) The role of religion and spirituality in cancer care: An umbrella review of the literature. Surg Oncol 42:101389. 10.1016/j.suronc.2020.05.004 34103240 10.1016/j.suronc.2020.05.004

[7] Canada AL, Fitchett G, Murphy PE, Stein K, Portier K, Crammer C, Peterman AH (2013) Racial/ethnic differences in spiritual well-being among cancer survivors. J Behav Med 36(5):441–453. 10.1007/s10865-012-9439-8 22752250 10.1007/s10865-012-9439-8

[8] Yates JS, Mustian KM, Morrow GR, Gillies LJ, Padmanaban D, Atkins JN, Issell B, Kirshner JJ, Colman LK (2005) Prevalence of complementary and alternative medicine use in cancer patients during treatment. Support Care Cancer 13(10):806–811. 10.1007/s00520-004-0770-7 15711946 10.1007/s00520-004-0770-7

[9] Jim HSL, Pustejovsky JE, Park CL, Danhauer SC, Sherman AC, Fitchett G, Merluzzi TV, Munoz AR, George L, Snyder MA, Salsman JM (2015) Religion, spirituality, and physical health in cancer patients: a meta-analysis. Cancer 121(21):3760–3768. 10.1002/cncr.29353 26258868 10.1002/cncr.29353 PMC4618080

[10] Salsman JM, Pustejovsky JE, Jim HS, Munoz AR, Merluzzi TV, George L, Park CL, Danhauer SC, Sherman AC, Snyder MA, Fitchett G (2015) A meta-analytic approach to examining the correlation between religion/spirituality and mental health in cancer. Cancer 121(21):3769–3778. 10.1002/cncr.29350 26258536 10.1002/cncr.29350 PMC4618157

[11] Sherman AC, Merluzzi T, Pustejovsky JE, Park CL, George L, Fitchett G, Jim HS, Munoz AR, Danhauer SC, Snyder MA, Salsman JM (2015) A meta-analytic review of religious or spiritual involvement and social health among cancer patients. Cancer 121(21). 10.1002/cncr.29352 10.1002/cncr.29352 PMC4618183 26258730

[12] Canada AL, Murphy PE, Stein K, Alcaraz KI, Leach CR, Fitchett G (2023) Assessing the impact of religious resources and struggle on well-being: a report from the American Cancer Society’s Study of Cancer Survivors-I. J Cancer Surviv 7(2):360–369. 10.1007/s11764-022-01226-8 10.1007/s11764-022-01226-8 PMC10084782 35726114

[13] Schultz M, Meged-Book T, Mashiach T, Bar-Sela G (2017) Distinguishing between spiritual distress, general distress, spiritual well-being, and spiritual pain among cancer patients during oncology treatment. J Pain Symptom Manage 54(1):66–73. 10.1016/j.jpainsymman.2017.03.018 28533159 10.1016/j.jpainsymman.2017.03.018

[14] Sprik P, Keenan AJ, Boselli D, Cheeseboro S, Meadors P, Grossoehme D (2021) Feasibility and acceptability of a telephone-based chaplaincy intervention in a large, outpatient oncology center. Support Care Cancer 29:1275–1285. 10.1007/s00520-018-4447-z 32623520 10.1007/s00520-020-05598-4 PMC7334628

[15] Damen A, Raijmakers NJH, van Roij J, Visser A, Beuken-Everdingen MVD, Kuip E, van Laarhoven HWM, van Leeuwen-Snoeks L, van der Padt-Pruijsten A, Smilde TJ, Leget C, Fitchett G (2022) Spiritual well-being and associated factors in Dutch patients with advanced cancer. J Pain Symptom Manage 63(3):404–414. 10.1016/j.jpainsymman.2021.10.004 34656652 10.1016/j.jpainsymman.2021.10.004

[16] Pérez-Cruz PE, Langer P, Carrasco C, Bonati P, Batic B, Tupper Satt L, Gonzalez Otaiza M (2019) Spiritual pain is associated with decreased quality of life in advanced cancer patients in palliative care: an exploratory study. J Palliat Med 22(6):663–669. 10.1089/jpm.2018.0340 30649985 10.1089/jpm.2018.0340

[17] Delgado-Guay MO, Chisholm G, Williams J, Frisbee-Hume S, Ferguson AO, Bruera E (2016) Frequency, intensity, and correlates of spiritual pain in advanced cancer patients assessed in a supportive/palliative care clinic. Palliat Support Care 14(4):341–348. 10.1017/S147895151500108X 26481034 10.1017/S147895151500108X

[18] Fitchett G, Yao Y, Emanuel LL, Guay MOD, Handzo G, Hauser J, Kittelson S, O’Mahony S, Quest T, Rabow M, Schoppee TM, Solomon S, Wilkie DJ, Chochinov HM (2024) Examining moderation of dignity therapy effects by symptom burden or religious/spiritual struggles. J Pain Symptom Manage 67(4):e333–e340. 10.1016/j.jpainsymman.2024.01.003 38215893 10.1016/j.jpainsymman.2024.01.003 PMC10939845

[19] Boscaglia N, Clarke DM, Jobling TW, Quinn MA (2005) The contribution of spirituality and spiritual coping to anxiety and depression in women with a recent diagnosis of gynecological cancer. Int J Gynecol Cancer 15(5):755–761. 10.1111/j.1525-1438.2005.00248 16174220 10.1111/j.1525-1438.2005.00248.x

[20] King SD, Fitchett G, Murphy PE, Pargament KI, Martin PJ, Johnson RH, Harrison DA, Loggers ET (2017) Spiritual or religious struggle in hematopoietic cell transplant survivors. Psychooncology 26(2):270–277. 10.1002/pon.4029 26567771 10.1002/pon.4029

[21] Manning-Walsh J (2005) Spiritual struggle: effect on quality of life and life satisfaction in women with breast cancer. J Holist Nurs 23(2):120–140. 10.1177/0898010104272019 15883461 10.1177/0898010104272019

[22] Hebert R, Zdaniuk B, Schulz R, Scheier M (2009) Positive and negative religious coping and well-being in women with breast cancer. J Palliat Med 12(6):537–545. 10.1089/jpm.2008.0250 19508140 10.1089/jpm.2008.0250 PMC2789454

[23] Sherman AC, Plante TG, Simonton S, Latif U, Anaissie EJ (2009) Prospective study of religious coping among patients undergoing autologous stem cell transplantation. J Behav Med 32(1):118–128. 10.1007/s10865-008-9179-y 18855130 10.1007/s10865-008-9179-y

[24] Delgado-Guay MO, Parsons HA, Hui D, De la Cruz MG, Thorney S, Bruera E (2013) Spirituality, religiosity, and spiritual pain among caregivers of patients with advanced cancer. Am J Hosp Palliat Care 30(5):455–461. 10.1177/1049909112458030 22952129 10.1177/1049909112458030

[25] Schultz M, Lulav-Grinwald D, Bar-Sela G (2014) Cultural differences in spiritual care: findings of an Israeli oncologic questionnaire examining patient interest in spiritual care. BMC Palliat Care 13(1):19. 10.1186/1472-684X-13-19 24708816 10.1186/1472-684X-13-19 PMC4108186

[26] Perez SEV, Maiko S, Burke ES, Slaven JE, Johns SA, Smith OJ, Helft PR, Kozinski K, Torke AM (2022) Spiritual care assessment and intervention (SCAI) for adult outpatients with advanced cancer and caregivers: a pilot trial to assess feasibility, acceptability, and preliminary effects. Am J Hosp Palliat Care 39(8):895–906. 10.1177/10499091211042860 34467769 10.1177/10499091211042860 PMC8928229

[27] Cadge W (2013) Paging God: religion in the halls of medicine. University of Chicago Press

[28] Handzo G, Hughes B, Bowden J, Kelly B, Lynch J, Mercier M, Pavlantos C, Rothstein H, Tuttle M (2022) Chaplaincy in the outpatient setting-getting from here to there. J Health Care Chaplain 28(2):194–207. 10.1080/08854726.2020.1818359 32981466 10.1080/08854726.2020.1818359

[29] Tartaglia A, Corson T, White KB, Charlescraft A, Jackson-Jordan E, Johnson T, Fitchett G (2024) Chaplain staffing and scope of service: benchmarking spiritual care departments. J Health Care Chaplain 30(1):1–18. 10.1080/08854726.2022.2121579 36102782 10.1080/08854726.2022.2121579

[30] Sprik PJ, Vanderstelt H, Valenti-Hein C, Denton J, Ashton D (2024) Chaplain interventions and outcomes in outpatient settings: a scoping review. J Health Care Chaplain 5:1–23. 10.1080/08854726.2024.2357042 10.1080/08854726.2024.2357042 38836429

[31] Kestenbaum A, Shields M, James J, Hocker W, Morgan S, Karve S, Rabow MW, Dunn LB (2017) What impact to chaplains have? A pilot study of spiritual AIM for advanced cancer patients in outpatient palliative care. J Pain Symptom Manage 54(5):707–714. 10.1016/j.jpainsymman.2017.07.027 28736103 10.1016/j.jpainsymman.2017.07.027 PMC5650916

[32] Piderman KM, Radecki Breitkopf C, Jenkins SM, Ingram C, Sytsma TT, Lapid MI, Tata BS, Chatterjee K, Egginton JS, Jatoi A (2020) Hearing and heeding the voices of those with advanced illnesses. J Palliat Care 35(4):248–255. 10.1177/0825859720928623 32466734 10.1177/0825859720928623

[33] Muehlhausen BL, Chappelle C, DeLaney A, Peacock D, Stratton RG, Fitchett G (2024) Providing spiritual care to cancer patients in the outpatient context: a pilot study. J Health Care Chaplain 30(3):153–166. 10.1080/08854726.2023.2266303 37811644 10.1080/08854726.2023.2266303

[34] Grady MP (1998) Qualitative and action research: a practitioner handbook. Phi Delta Kappa Educational Foundation, Bloomington

[35] Braun V, Clarke V (2006) Using thematic analysis in psychology. Qual Res Psych 3(2):77–101

[36] Keogh M, Sharma V, Myerson SL, Marin DB (2017) The Chi Cart ministry. Nurs Manag 48(8):32–38. 10.1097/01.NUMA.0000521574.35431.50 10.1097/01.NUMA.0000521574.35431.50 28749803

[37] Riba M, Donovan KA, Anderson B et al (2019) Distress management version 3.2019. J Natl Compr Canc Netw 17(10):1229–1249. 10.6004/jnccn.2019.0048 31590149 10.6004/jnccn.2019.0048 PMC6907687

[38] Deshields T, Kracen A, Nanna S, Kimbro L (2016) Psychosocial staffing at National Comprehensive Cancer Network member institutions: data from leading cancer centers. Psychooncology 25(2):164–169. 10.1002/pon.3826 25963109 10.1002/pon.3826

[39] Deshields T, Zebrack B, Kennedy V (2013) The state of psychosocial services in cancer care in the United States. Psychooncology 22(3):699–703. 10.1002/pon.3057 22354821 10.1002/pon.3057

[40] Handzo G, Bowden JM, King S (2019) The evolution of spiritual care in the NCCN distress management guidelines. J Natl Compr Canc Netw 17(10):1257–1261. 10.6004/jnccn.2019.7352 31590159 10.6004/jnccn.2019.7352

[41] Tartaglia A, White KB, Corson T, Charlescraft A, Johnson T, Jackson-Jordan B, Fitchett G (2024) Supporting staff: The role of health care chaplains. J Health Care Chaplain 30(1):60–73. 10.1080/08854726.2022.2154107 36520544 10.1080/08854726.2022.2154107

[42] White KB, Galchutt P, Collier K, Szilagyi C, Fitchett G (2024) Chaplains’ reports of integration in community health initiatives: a qualitative study. J Health Care Chap. 10.1080/08854726.2024.2401742 10.1080/08854726.2024.2401742 39294900

[43] Robinson MR, Thiel MM, Shirkey K, Zurakowski D, Meyer EC (2016) Efficacy of training interprofessional spiritual care generalists. J Health Care Chap 19(8):814–821. 10.1089/jpm.2015.0373 10.1089/jpm.2015.0373 27115716

[44] Puchalski C, Ferrell BR, Borneman T, DiFrances RC, Haythorn T, Jacobs C (2022) Implementing quality improvement efforts in spiritual care: outcomes from the interprofessional spiritual care education curriculum. J Health Care Chap 28(3):431–442. 10.1080/08854726.2021.1917168 10.1080/08854726.2021.1917168 34396929

[45] Bergen N, Labonté R (2020) “Everything is perfect, and we have no problems”: detecting and limiting social desirability bias in qualitative research. Qual Health Res 30(5):783–792. 10.1177/1049732319889354 31830860 10.1177/1049732319889354

